# Physiological Ecology of Microorganisms in Subglacial Lake Whillans

**DOI:** 10.3389/fmicb.2016.01705

**Published:** 2016-10-27

**Authors:** Trista J. Vick-Majors, Andrew C. Mitchell, Amanda M. Achberger, Brent C. Christner, John E. Dore, Alexander B. Michaud, Jill A. Mikucki, Alicia M. Purcell, Mark L. Skidmore, John C. Priscu, The WISSARD Science Team

**Affiliations:** ^1^Department of Land Resources and Environmental Sciences, Montana State UniversityBozeman, MT, USA; ^2^Department of Geography and Earth Sciences, Aberystwyth UniversityAberystwyth, UK; ^3^Department of Biological Sciences, Louisiana State UniversityBaton Rouge, LA, USA; ^4^Department of Microbiology and Cell Science, University of FloridaGainesville, FL, USA; ^5^Biodiversity Institute, University of FloridaGainesville, FL, USA; ^6^Department of Microbiology, University of TennesseeKnoxville, TN, USA; ^7^Department of Earth Sciences, Montana State UniversityBozeman, MT, USA; ^8^http://www.wissard.org

**Keywords:** subglacial environments, Antarctica, subglacial lake, microbial energetics, microbial physiological ecology, thermodynamics, thymidine and leucine incorporation, oxygen consumption

## Abstract

Subglacial microbial habitats are widespread in glaciated regions of our planet. Some of these environments have been isolated from the atmosphere and from sunlight for many thousands of years. Consequently, ecosystem processes must rely on energy gained from the oxidation of inorganic substrates or detrital organic matter. Subglacial Lake Whillans (SLW) is one of more than 400 subglacial lakes known to exist under the Antarctic ice sheet; however, little is known about microbial physiology and energetics in these systems. When it was sampled through its 800 m thick ice cover in 2013, the SLW water column was shallow (~2 m deep), oxygenated, and possessed sufficient concentrations of C, N, and P substrates to support microbial growth. Here, we use a combination of physiological assays and models to assess the energetics of microbial life in SLW. In general, SLW microorganisms grew slowly in this energy-limited environment. Heterotrophic cellular carbon turnover times, calculated from ^3^H-thymidine and ^3^H-leucine incorporation rates, were long (60 to 500 days) while cellular doubling times averaged 196 days. Inferred growth rates (average ~0.006 d^−1^) obtained from the same incubations were at least an order of magnitude lower than those measured in Antarctic surface lakes and oligotrophic areas of the ocean. Low growth efficiency (8%) indicated that heterotrophic populations in SLW partition a majority of their carbon demand to cellular maintenance rather than growth. Chemoautotrophic CO_2_-fixation exceeded heterotrophic organic C-demand by a factor of ~1.5. Aerobic respiratory activity associated with heterotrophic and chemoautotrophic metabolism surpassed the estimated supply of oxygen to SLW, implying that microbial activity could deplete the oxygenated waters, resulting in anoxia. We used thermodynamic calculations to examine the biogeochemical and energetic consequences of environmentally imposed switching between aerobic and anaerobic metabolisms in the SLW water column. Heterotrophic metabolisms utilizing acetate and formate as electron donors yielded less energy than chemolithotrophic metabolisms when calculated in terms of energy density, which supports experimental results that showed chemoautotrophic activity in excess of heterotrophic activity. The microbial communities of subglacial lake ecosystems provide important natural laboratories to study the physiological and biogeochemical behavior of microorganisms inhabiting cold, dark environments.

## Introduction

Subglacial aquatic habitats, including lakes, streams, and water saturated sediments, reside beneath polar ice sheets and mountain glaciers (Skidmore et al., 2000; Tranter et al., 2005; Christner et al., 2006, 2014; Gaidos et al., 2009; Lanoil et al., 2009; Mikucki et al., 2009; Dieser et al., 2014). The ~400 subglacial lakes documented beneath the ~1–4 km thick Antarctic ice sheet (Siegert et al., 2015) hold an estimated 10^21^ microbial cells, comprising 1600 teragrams of cellular C in ~10,000 km^3^ of liquid water (Priscu et al., 2008). While photosynthesis is the primary source of energy in the sunlit biosphere (Behrenfeld et al., 2005), subsurface environments beneath Antarctic ice sheets are aphotic. Hence, photosynthetic primary production cannot provide the basis for these food webs. With the exception of deep sea hydrothermal vents, where strong chemical gradients support high rates of microbial activity (e.g., Orcutt et al., 2011), communities lacking direct photosynthetic inputs typically have low metabolic rates (Røy et al., 2012). Heterotrophic activity in subglacial waters may be supported by low inputs of organic C from melting glacial ice (Antarctic ice sheet dissolved organic C ≈ 0.15 mg L^−1^; Hood et al., 2015), or from organic matter stored in relict marine sediments beneath the ice sheet (Wadham et al., 2012). Most microbial energy is thought to be supplied via chemolithoautotrophic metabolism (e.g., Boyd et al., 2011, 2014), which has been hypothesized to drive subglacial environments toward anoxia (Wadham et al., 2010).

In contrast to the marginal regions of northern hemisphere ice sheets, Antarctic subglacial aquatic environments do not receive direct inputs of surface melt (Skidmore, 2011; Willis et al., 2015). As such, these ice-sealed Antarctic subglacial aquatic environments are isolated from direct surface contact and ideal for the study of dark microbial ecosystem processes. Subglacial Lake Whillans (SLW) in West Antarctica, the first Antarctic subglacial lake to be explored and sampled directly (Tulaczyk et al., 2014), is part of a continuum of hydrologically active subglacial lakes along the Siple Coast (Fricker et al., 2007). Active subglacial lakes are characterized by their fluctuations in lake volume over time (Smith et al., 2009). Outflow from SLW flows beneath the Whillans Ice Stream and drains into the ocean beneath the Ross Ice Shelf, and the lake is refilled by periodic inflow from upstream (Siegfried et al., 2014); the ice surface rises and falls with the lake level (Fricker et al., 2007). Microbiological analyses of water and sediment samples from SLW revealed the presence of active microbial communities and evidence for an ecosystem driven by chemosynthetic production (Christner et al., 2014; Mikucki et al., 2015; Achberger et al., 2016) and by organic matter and nutrients contained in relict marine sediments beneath the West Antarctic Ice Sheet (Scherer et al., 1998; Wadham et al., 2012; Michaud et al., 2016). This evidence, coupled with the connectivity of SLW to the hydrological network of this region (Fricker et al., 2007), suggests microbial life is widespread beneath the ice sheet.

Despite the evidence for widespread subglacial microbial life, little is known about the long term sustainability of microbial ecosystems under ice, or how they derive their energy. Most studies of microbial metabolism under glaciers have focused on chemolithotrophic activity, which can be supported by the oxidation or reduction of nitrogen, iron, and/or sulfur (Mikucki et al., 2009; Boyd et al., 2011, 2014; Mitchell et al., 2013), with comparatively little attention paid to the heterotrophic components of the community. Methane is likely an important source of carbon and energy under the Greenland (Dieser et al., 2014) and Antarctic (Wadham et al., 2012) ice sheets; methanotrophy supports rates of organic carbon production in SLW that match rates of chemoautotrophic carbon fixation (Christner et al., 2014; Michaud, 2016). West Antarctica, the site of SLW, is underlain by relict marine sediments that may be a source of dissolved organic matter and nutrients (Wadham et al., 2012). The contemporary production of dissolved organic matter under ice sheets (e.g., Christner et al., 2014) is likely the result of release by chemoautotrophic and/or heterotrophic microbial cells, and/or the degradation of necromass. Such chemoautotrophically produced dissolved organic matter may be substantial in concentration (Kawasaki et al., 2013), but less labile in nature than photosynthetically produced carbon (Ogawa et al., 2001). Low-lability organic matter can be mineralized by heterotrophic microorganisms, but mineralization occurs over long time scales (years to decades; Carlson, 2002), with low metabolic rates (e.g., 10^−5^ to 10 ^−3^ fmol C cell^−1^ d^−1^) compared to surface environments (e.g., 0.1 to 10 fmol C cell^−1^ d^−1^; Jørgensen, 2011).

The average C:N ratio of microbial biomass in the oceans is 6.6 (Redfield et al., 1963) while that of particulate organic matter in SLW is 65.4, implying N-deficiency (Christner et al., 2014). This depletion of N relative to C is consistent with evidence of N-limitation along with an active N-cycle in other subglacial environments, such as the Robertson Glacier in Canada (Boyd et al., 2011), and suggests that microbial activity in subglacial environments may be constrained, at least in part, by the availability of fixed N. In addition to nutrient limitation, microorganisms in SLW, where water exists at the pressure freezing point (Fisher et al., 2015), need to tolerate near-zero degree temperatures. Low temperature can negatively impact growth (Pomeroy and Deibel, 1986) and decrease the free energy gained from metabolic reactions (LaRowe and Amend, 2015), suggesting that subzero temperatures in subglacial aquatic environments may impose additional limitations on microbial anabolism and catabolism beneath Antarctic ice (Price and Sowers, 2004).

Here, we use a combination of physiological assays and models to assess the energetics of, and the geochemical signatures potentially imparted by, microbial life in SLW. Our results show that heterotrophic growth in the SLW water column is slow and inefficient in spite of high calculated energy yields for heterotrophic metabolism, but that biological activity in the water column and surface sediments leads to a drawdown of dissolved oxygen; this O_2_ consumption is largely the result of water column chemoautotrophic activity. The drawdown of oxygen has physiological, biogeochemical, and bioenergetic consequences for the lake ecosystem, and may help to constrain estimates of hydrologic residence time beneath this region of the West Antarctic Ice Sheet. The modeled oxygen drawdown led us to posit that switching between oxic and anoxic conditions and associated aerobic and anaerobic microbial metabolisms plays a key role in elemental cycling in the SLW water column. We applied thermodynamic calculations to explore this hypothesis.

## Methods

### Sample collection

The lake was sampled through a ~0.6 m diameter borehole created through the 800 m thick ice cover with a hot water drilling system that was effective in removing and killing microorganisms present in the drilling water (Priscu et al., 2013; Christner et al., 2014; Tulaczyk et al., 2014). Three discrete 10 L water samples were collected at mid-depth in the ~2.2 m water column using a 3% hydrogen peroxide-cleaned Niskin bottle on January 28 (cast 1; C1), 30 (cast 2; C2), and 31 (cast 3; C3), 2013 (Tulaczyk et al., 2014) and returned to an on-site laboratory for processing. Samples for microbiological characterization were decanted through an acid-washed hose into acid-washed (1% hydrochloric acid; rinsed 5X with ultra-pure water) and autoclaved opaque high density polyethylene (HDPE) bottles.

### Determination of cell sizes and morphology

The average cell size and relative abundances of cell morphologies were determined using samples stained with SYBR Gold-nucleic acid stain as described by Christner et al. (2014). Digital images were captured at 1000x magnification using a Nikon Eclipse 80i epifluorescence microscope equipped with a Metal Halide lamp, a 450–490 excitation filter, and a digital CCD Camera (Retiga 2000R Color Cooled). Photographs were analyzed using ImageJ v. 2.0 (Schindelin et al., 2012), and a minimum of 300 cells were measured per sample. The surface area of each cell was calculated using ImageJ v. 2.0 software (Schindelin et al., 2012) built-in tools, and converted to the spherical equivalent particle diameter to normalize for differences in cell morphologies (Jackson, 2005).

### Microbial production, growth, and carbon turnover

Rates of heterotrophic microbial production in SLW sample casts 1, 2, and 3 (i.e., based on [^3^H]-thymidine) and 1 and 3 ([^3^H]-leucine) are from Christner et al. (2014). These data were derived from endpoint incubations conducted using samples collected from three hydrocasts (Tulaczyk et al., 2014) that were amended with [^3^H] methyl-thymidine (specific activity 20 Ci mmol^−1^) and [^3^H]-leucine (specific activity 84 Ci mmol^−1^) to a final added substrate concentration of 20 nM, followed by incubating at 4°C in the dark, as described by Christner et al. (2014). Heterotrophic microbial production in cast 2 (^3^H-leucine) was determined as the slope of the line when leucine incorporation is plotted vs. the incubation time, from incubations conducted as described by Christner et al. (2014), except that the reactions in duplicate vials were terminated by the addition of 100 μl of cold 100% w/v trichloroacetic acid (TCA; 5% final concentration) at 0, 20, 65, 80, 137, and 161 h. Samples were then centrifuged and washed with cold 5% w/v TCA and cold 80% v/v ethanol to remove unincorporated label. The pellet was dried overnight at ~25°C and amended with 1 ml of Cytoscint ES (MP Biomedicals). The radioactivity incorporated in the pellet was determined using a calibrated liquid scintillation counter. Rates of thymidine and leucine incorporation at incubation temperature were corrected to *in situ* temperature using the energy of activation determined in our temperature experiments (see below) as described by Takacs and Priscu (1998).

Leucine (Leu) and thymidine (TdR) incorporation were converted to cell production and carbon production using the following conversion factors: 1.4 × 10^17^ cells mol^−1^ leucine incorporated (Chin-Leo and Kirchman, 1988) or 2.0 x 10^18^ cells mol^−1^ thymidine incorporated (Bell, 1993; Takacs and Priscu, 1998) and 11 fg C cell^−1^ (Kepner et al., 1998). Cell production (cells ml^−1^ d^−1^) was divided by cell concentration (Christner et al., 2014) to determine cell specific growth rate (day^−1^). Carbon turnover rate was calculated by dividing bacterial production (nmol C L^−1^ d^−1^) by cellular carbon (nmol C L^−1^). Doubling times were calculated assuming exponential growth as ln2/cell specific growth rate e.g., (Crump et al., 2004).

### Substrate kinetics of leucine incorporation

The substrate kinetics of leucine incorporation were determined by amending the samples with 2000, 5000, 8000, 10,000, 12,000, 18,000, and 20,000 pmol [^3^H]-leucine L^−1^ (five replicates per concentration, with TCA-killed controls (5% final concentration) at 2000, 12,000, and 20,000 pmol leucine L^−1^) and incubating at 2–4°C as described above. The maximum incorporation rate at saturating substrate concentration (V_max_) and the half saturation coefficient (K_*t*_; i.e., the substrate concentration where incorporation velocity = V_max_/2) were obtained by direct non-linear fit of the data with the Marquardt algorithm (Marquardt, 1963) assuming incorporation followed Michaelis-Menten kinetics.

### Nutrient deficiency experiments

A nutrient bioassay was conducted by dispensing 200 ml of sample from cast 2 into an acid (10% HCl) cleaned, autoclaved HDPE bottle and mixing well. The sample was then divided among seven 60 ml bottles. Six bottles were amended with either C (glucose, final concentration 100 uM), N (NH_4_Cl, final concentration 20 uM), P (KH_2_PO_4_, final concentration 2 μmol L^−1^), C+N, N+P, or C+N+P. The seventh bottle served as an unamended control. Three 1.5 ml aliquots were immediately withdrawn from each bottle and placed in 2 ml sterile micro centrifuge tubes (time zero). Each tube was amended with [^3^H]-leucine and incubated as described previously to determine rates of leucine incorporation at time zero. All bottles were then incubated at 2–4°C in the dark and three aliquots were withdrawn from each bottle and incubated with [^3^H]-leucine after bottles had incubated for 23, 43, 157, and 187 h.

In order to account for unequal sample size (two unamended control sample vials were lost at the final time point) and to account for changes through time prior to determining the effect of nutrient amendment, we used a Type III Sum of Squares (ANOVA) test to analyze the results of the bioassay, followed with directed comparisons (contrasts) between the control and each nutrient amendment to minimize the chance of a Type I error. Statistical calculations were run using SAS (version 9.4).

### Leucine incorporation as a function of temperature

Samples (1.5 ml) from cast 2 were amended with [^3^H]-leucine and incubated at discrete temperatures between 1.9 and 10.3°C. An aluminum block with 12 rows of incubations wells (3 wells per row + 1 thermometer well per row) was affixed to a low temperature circulating water bath on one end, and a high temperature circulating water bath on the other (Thomas et al., 1963). The incubation and thermometer wells were filled with deionized water and the system was allowed to equilibrate for ~24 h before the experiment was initiated. Six [^3^H]-leucine amended (20 nmol L^−1^ final concentration) 1.5 ml samples (three live and three TCA-killed controls) were placed in the incubation wells corresponding to a given temperature (1.9, 2.6, 3.4, 4.2, 5.0, 5.7, 6.5, 7.3, 8.0, 8.8, 9.6, and 10.3°C). Temperature was monitored throughout the incubation with a digital thermometer. After determination of leucine incorporation rates, the Q_10_ and energy of activation (E_a_) were determined using an Arrhenius plot.

### Heterotrophic respiration

Heterotrophic respiration was measured by adding 60 ml of water sample (Niskin cast 1) to an autoclaved amber HDPE bottle (Nalgene) followed by the addition of uniformly labeled ^14^C-L-leucine (final added leucine concentration 60 nmol L^−1^; final activity 0.0180 μCi ml^−1^; del Giorgio et al., 2011). Five-milliliter aliquots of the radiolabeled sample were added to autoclaved 25 ml glass side arm flasks (6 live and 6 TCA killed controls; 250 μL of cold 100% TCA). The top of the flask was sealed with a butyl rubber septum holding a small basket containing a folded GF/C filter that was suspended above the aqueous phase (Christner et al., 2006); the sidearm was sealed with a butyl rubber septum. Following incubation in the dark for 105 h at 2–4°C, the incubations were terminated by injecting cold 100% TCA (final concentration 5%) into the sample through the sidearm, lowering the pH to ≤ 2. β-phenylethylamine (100 μL; Sigma, catalog number P2641) was added to the GF/C filter through the septum with a needle and syringe to trap respired CO_2_. Killed samples were incubated at ~25°C for 24 h with occasional gentle swirling to liberate CO_2_ from the aqueous phase. Cellular ^14^C incorporation was determined on the liquid fraction following filtration onto 0.2 μm polycarbonate filters and rinsing with 5% TCA. The GF/C (respired fraction) and polycarbonate filters (incorporated fraction) were placed in 20 ml scintillation vials followed by the addition of 10 ml of Cytoscint-ES and the ^14^C activity was determined using a calibrated scintillation counter.

Bacterial growth efficiency (the percentage of biomass produced per unit of carbon consumed) was calculated from the leucine respiration data as follows: ((Leu incorporation)/(Leu incorporation + Leu respiration)) × 100. ^14^C-leucine incorporation and respiration were converted to units of carbon as described above, and total heterotrophic bacterial carbon demand was calculated as the sum of carbon respired and carbon fixed into biomass.

### Oxygen budget

The SLW water column was 2 m deep, mixed, and oxic at the time of sampling (Christner et al., 2014; Tulaczyk et al., 2014). The supply of O_2_ to SLW was determined by assuming that subglacial water entering SLW (Siegfried et al., 2016) had an O_2_ concentration equal to that in the water column at the time of sampling (Table S1), and that atmospheric O_2_ from melting meteoric ice was the only source (Christner et al., 2014). The total annual input of O_2_ (*O*_*t*_ = km^3^ O_2_ y^−1^) to SLW was determined from Equations (1) through (3):

(1) $$\begin{array}{l} O_{t}=O_{m}+\;O_{f} \end{array}$$

Where *O*_*m*_ is the O_2_ from ice melt (km^3^ O_2_ y^−1^), defined as:

(2) $$\begin{array}{l} O_{m}=\left(GF\;\times \;OF\right)\times \left(M\;\times \;A\right) \end{array}$$

where *GF* is the gas fraction in meteoric ice (10%), *OF* is the fraction of oxygen in the modern atmosphere (20.95%), *M* is the ice melt rate over SLW (1.8 cm y^−1^; Fisher et al., 2015) converted to km y^−1^, and *A* is the surface area of SLW (60 km^2^; (Fricker and Scambos, 2009). *O*_*f*_ is the O_2_ from water inflow from upstream (km^3^ O_2_ y^−1^), defined as:

(3) $$\begin{array}{l} O_{f}=F\;\times \;C \end{array}$$

where *F* is the volume of water that entered SLW during the year leading up to sampling (0.007 km^3^ H_2_O y^−1^; calculated from data presented by Siegfried et al., 2016) and *C* is the concentration of O_2_ in SLW (71.9 μmol L^−1^; Christner et al., 2014; converted to km^3^ O_2_ per km^3^ of H_2_O using conversion 10^12^ L km^−3^).

The resulting volume of O_2_ (*O*_*t*_) was then converted to moles of O_2_ using 22.4 L mol^−1^ of gas (volume of an ideal gas at standard temperature and pressure), and the converted value was used in the oxygen budget.

The biological demand for O_2_ was determined by stoichiometrically summing the amount of O_2_ associated with metabolic processes for which there was evidence in the water column and surface sediments of SLW (i.e., heterotrophy, chemoautotrophy, and methane oxidation; Christner et al., 2014; Michaud, 2016) over the entire lake volume (0.132 km^3^; based on a square-sided basin with 60 km^2^ surface area; Fricker and Scambos, 2009) with 2.2 m water column depth (Christner et al., 2014).

The specific O_2_ demand estimate for nitrification assumed that all dark inorganic carbon fixation measured in the SLW water column (32.9 ng C L^−1^ d^−1^; Christner et al., 2014) was chemoautotrophic and a product of nitrification. There is evidence that rates of chemosynthesis are higher at the sediment water interface (Mikucki et al., 2015); however, here we assume that the rate of carbon fixation was constant with depth over the area of the lake. The O_2_ demand was calculated assuming 0.1 moles of carbon fixed for every mole of ammonium oxidized (Berg et al., 2015) and a stoichiometry for complete nitrification of NH${}_{4}^{+}$ to NO${}_{3}^{-}$ of 2 mol of O_2_ per mol of nitrogen oxidized.

The O_2_ demand for heterotrophic respiration in the water column was calculated by converting the leucine respiration rate to carbon (described above), and assuming a respiratory quotient (RQ) of 0.75 moles of CO_2_ per mole of O_2._ Heterotrophic respiratory quotients can vary widely, but net autotrophic freshwater lakes have been shown to have RQ < 1 (Berggren et al., 2012), and respiration of amino acids and organic acids (the labile organic compounds known to be present in SLW; (Christner et al., 2014) have also been associated with RQ < 1 (Dilly, 2001). Water column O_2_ demand for heterotrophic C incorporation was determined by converting the rate of leucine-based C incorporation to oxygen based on biomass stoichiometry of 138 O_2_:106 C (Redfield et al., 1963). Surface sediment oxygen demand was derived from the sum of the rates of leucine incorporation converted to carbon (Christner et al., 2014) and respiration estimated from the water column growth efficiency (see above). The sediment estimates assumed the same stoichiometry as for water column heterotrophic respiration. The oxygen demand for methane oxidation was determined using estimated rates of methane oxidation (Michaud, 2016). We used the stoichiometry for methane oxidation shown in Table S2, and assumed that the fraction of methane incorporated into biomass was 0.5 (a median value across habitats; Shelley et al., 2014; Trimmer et al., 2015), resulting in 1.5 mol O_2_ consumed per mol of CH_4_ oxidized.

### Thermodynamic calculations

To examine free energy changes associated with the oxidation of inorganic chemical species in SLW, we determined and ranked the chemical affinity of coupled oxidation-reduction reactions for two redox scenarios. The amount of energy yielded from each reaction depended on the concentrations of reactants and products as well as the standard state thermodynamic conditions of the reaction, as described below. The chemical affinity (*A*_*r*_) is the maximum amount of energy that can be obtained for a reaction in a given environment and is calculated based on the change in the overall Gibbs energy under non-equilibrium conditions (Δ*G*${}_{r}^{{}^{\circ}}$) (Amend and Shock, 2001; Shock et al., 2010) with the following expression:

(4) $$\begin{array}{l} A_{r}=RT\ln\;\left(K_{r}/Q_{r}\right) \end{array}$$

*K*_*r*_ is the calculated equilibrium constant for the reaction, which is derived from Δ*G*${}_{r}^{{}^{\circ}}$ of the reaction according to Δ*G*${}_{r}^{{}^{\circ}}$ = $G_{f}^{o}$ products— $G_{f}^{o}$ reactants, where $G_{f}^{o}$ is the standard Gibbs energies of formation for the products and reactants (Stumm and Morgan, 1996). *K*_*r*_ is given by;

(5) $$\begin{array}{l} K_{r}=e^{-\Delta G_{r}^{o}/RT} \end{array}$$

where *R* is the gas constant 0.008314 kJ mol^−1^, and *T* is SLW temperature in Kelvin (−0.5°C = 272.62 K; Stumm and Morgan, 1996). Thermodynamic values were derived from Amend and Shock (2001) using values for 2°C, the closest available values for the temperature of SLW (−0.5°C); the temperature impact on $\Delta G_{r}^{o}$ and resulting *K*_*r*_ values will be small (Amend and Shock, 2001).

*Q*_*r*_ is the activity product for the reaction, determined as

(6) $$\begin{array}{l} Q_{r}=\prod_{i}{\left(a_{i}\right)}^{V_{i,r}} \end{array}$$

where *a*_*i*_ represents the activity of the ith compound in the reaction raised to its stoichiometric coefficient in the rth reaction, *v*_*i, r*_, which is positive for products and negative for reactants. Activities are calculated from molal concentrations (*m*_*i*_) using activity coefficients (γ_*i*_) and the relationship *a*_*i*_ = *m*_*i*_γ_*i*_ (Shock et al., 2010). These activities were determined using the PHREEQC geochemical model (Parkhurst and Appelo, 1999) using the measured SLW water chemistry (Table S1). The chemical affinities are expressed in per electron yields (*A*${}_{r}^{e-}$) and are also shown in terms of energy density, the energy per kg H_2_O (*A*${}_{r}^{kg}$), which scales the energy availability to the limiting reactant, calculated as

(7) $$\begin{array}{l} A_{r}^{kg}=|\frac{A_{r}}{v_{i}}|\left[i\right] \end{array}$$

where [*i*] refers to the concentration of the limiting electron donor or acceptor (LaRowe and Amend, 2014). This scaling of Gibbs energies has been shown to better correlate with actual microbial communities and metabolisms than the Gibbs energies normalized to moles of electrons transferred (LaRowe and Amend, 2014; Osburn et al., 2014).

To capture possible variations in lake redox potential, we calculated two potential geochemical scenarios for the SLW water column. The scenarios focused on redox couples that were geochemically plausible, as well as those indicated by potential metabolisms derived from microbial community data, which included aerobic organisms in addition to facultative aerobes, and strict anaerobes (Christner et al., 2014; Purcell et al., 2014; Achberger et al., 2016). Scenario A used the observed physicochemical measurements from the SLW water column: temperature, pH, redox (pE), and concentrations of acetate, formate, DIC, O_2_(aq), CH_4_(aq), SO${}_{4}^{2-}$, NO${}_{3}^{-}$, NO${}_{2}^{-}$, NH${}_{4}^{+}$, Cl^−^ and F^−^, Total dissolved Fe, Ca, Mg, Na, K, and P (Table S1). Redox sensitive elements that were measured as total dissolved elemental concentration (i.e., C, Fe) were assumed to be speciated to the redox states and species activities determined in PHREEQC. Conversely, ions measured in specific redox states (i.e., SO${}_{4}^{2-}$, NO${}_{3}^{-}$, NO${}_{2}^{-}$, NH${}_{4}^{+}$) were kept in their respective redox states by the model, and the species activities including these ions were calculated. Solid phase minerals pyrite (FeS_2_) and magnetite (Fe_3_O_4_), which can act as electron donors and acceptors, were detected in the lake sediments via x-ray diffraction (Hodson et al., 2016; Michaud et al., 2016), and defined in their standard states and thus with an activity of unity.

Scenario B simulated a more reduced SLW, where O_2_(aq) was set to 1 nM (anoxic marine zones are thought to have oxygen concentrations ≤ 3 nM; Ulloa et al., 2012) and pE was reduced to 2 (120 mV). This simulated mildly anoxic conditions. Dissolved O_2_ is typically in the nanomolar range or undetectable below 1.69 pE (100 mV) (e.g., Hargrave, 1972; Stumm and Morgan, 1996; Revsbech et al., 2009). All other values were kept as in Scenario A.

## Results

### Cell abundance and morphology

Microbial cell abundance in SLW was 1.3 × 10^5^ (±0.4 × 10^5^) cells ml^−1^, and a variety of morphologies were present (Christner et al., 2014). The cells observed were relatively small, with an average spherical equivalent diameter (Jackson, 2005) of 0.4 (± 0.1) μm. Cell populations were dominated by coccoid-shaped cells (59%), followed by rods (28%), spirochetes and curved rods (12%), and filaments (1%; data not shown).

### Heterotrophic activity

Rates of TdR incorporation ranged from 0.01 to 0.03 pmol TdR L^−1^ h^−1^, while rates of Leu incorporation ranged from 0.08 to 0.12 pmol Leu L^−1^ h^−1^, with the highest rates being measured in cast 2. These molar incorporation rates equated to a ratio of Leu:TdR = 3.6. Rates of carbon incorporation were also greatest in cast 2 and averaged 1.1 (± 0.5) nmol C l^−1^ d^−1^ and 0.29 (± 0.08) nmol C l^−1^ d^−1^, (thymidine and leucine, respectively; this study and Christner et al., 2014) (Table 1).

**Table 1:** **Heterotrophic substrate incorporation, turnover times, and growth rates determined from incubations with radiolabeled thymidine (TdR) and leucine (Leu)**.

	**Community incorporation (pmol TdR or Leu L^−1^ h^−1^)**		**Community incorporation (nmol C L^−1^ d^−1^)**		**Growth Rate (d^−1^)x 10^−3^**		**Doubling Time (days)**		**Cellular C Turnover Time (days)**	
	**TdR**	**Leu**	**TdR**	**Leu**	**TdR**	**Leu**	**TdR**	**Leu**	**TdR**	**Leu**
Cast 1	0.01	0.08	0.61	0.25	5.0	2.0	138	338	209	511
Cast 2	0.04	0.12	1.7	0.38	1.4	3.0	50	224	61	339
Cast 3	0.03	0.08	1.1	0.24	9.0	2.0	76	350	116	537
Average (*SD*)	0.03 (0.01)	0.09 (0.02)	1.1 (0.5)	0.29 (0.08)	9.0 (5.0)	2.0 (1.0)	88 (46)	304 (70)	129 (75)	462 (108)

Growth rates (d^−1^) averaged 0.009 (± 0.004; thymidine) and 0.002 (± 0.001; leucine), with a grand average of 0.006 (± 0.005); this equated to doubling times of 88 and 304 days for thymidine and leucine, respectively, with an average of 196 (Table 1). The average cellular C turnover times were 129 (± 75; thymidine) and 462 (± 108; leucine) days. The rate of C respiration rates (leucine-based) in SLW (1.7 nmol C L^−1^ d^−1^) exceeded incorporation rates from the same incubations (0.16 nmol C L^−1^ d^−1^; Vick-Majors, 2016) by a factor of 10.6, indicating low growth efficiency (8%). The time course incubation from cast 2 revealed that leucine incorporation rates remained linear during our period of incubation (up to 162 h; *r*^2^ = 0.96, data not shown).

### Substrate kinetics of leucine incorporation

**Figure 1:**
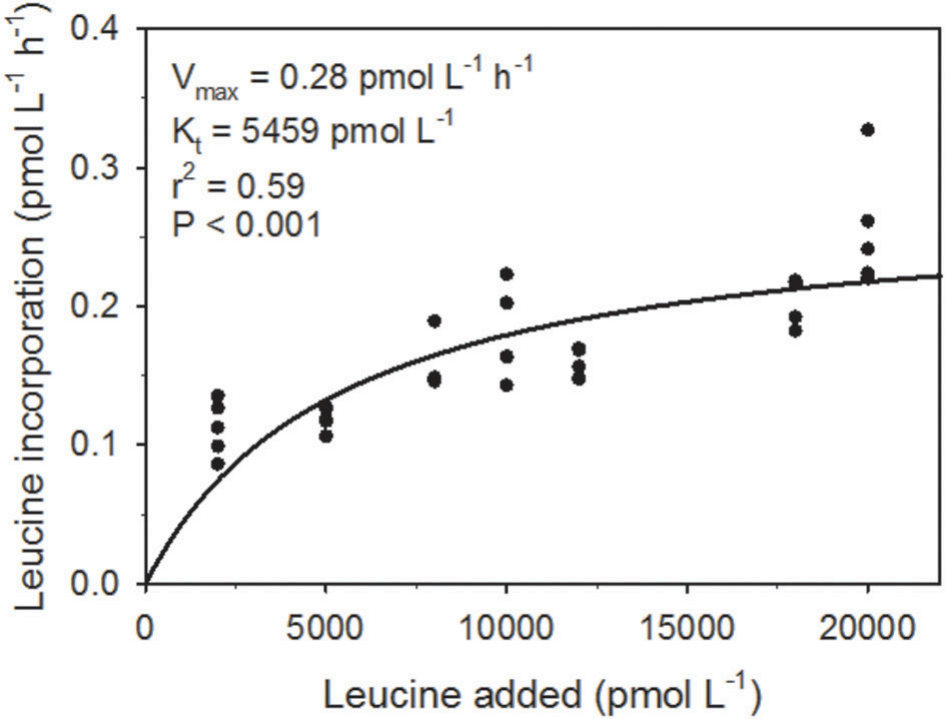
**The response of leucine incorporation to substrate concentration**. The maximum rate of incorporation at saturating substrate concentration (V_max_; pmol L^−1^ h^−1^) and the half-saturation concentration (K_*t*_; pmol L^−1^ h^−1^; the substrate concentration where the incorporation rate is equal to half of V_max_) were obtained by direct non-linear fit of the data with the Marquardt algorithm (Marquardt, 1963), assuming incorporation followed Michaelis-Menten kinetics.

The maximum potential leucine incorporation rate (V_*max*_) was 0.28 pmol leu L^−1^ h^−1^ and K_*t*_ (the concentration at which the incorporation rate = 0.5 × V_*max*_) was 5459 pmol leu L^−1^ (Figure 1). The average Leu incorporation rate (0.09 pmol leu L^−1^ h^−1^; Table 1) was ~3x lower than V_max_, while the maximum Leu incorporation rate (0.12 pmol leu L^−1^ h^−1^), which was determined from the same cast, was ~2x lower.

### Heterotrophic response to nutrient amendment and temperature

We used bottle incubation experiments to quantify the heterotrophic response to additions of organic C (glucose), inorganic N (NH_4_Cl), or inorganic P (KH_2_PO_4_), both individually and in combination.

**Figure 2:**
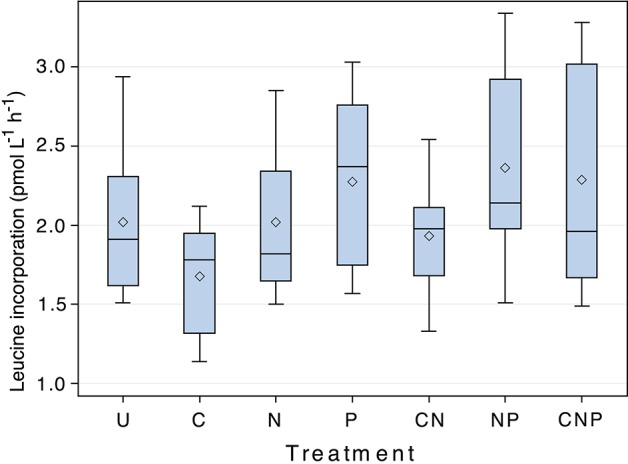
**The response of leucine incorporation to nutrient amendment**. Results of ANOVA analysis of the response of leucine incorporation to nutrient amendment, grouped by nutrient treatment. U = unamended control, C = Carbon, N = Nitrogen, P = Phosphorus. The mean and median for each treatment are indicated by the diamond and straight line, respectively. Each bar accounts for the variability associated with each treatment through time. Differences between treatments were significant (*F* = 23.02, *p* < 0.001, *d.f*. = 6).

After statistically accounting for changes in incorporation rates through time, nutrient amendments affected the rates of heterotrophic activity measured by leucine incorporation (*F* = 23.02, *p* < 0.001, *d.f*. = 6; Figure 2), but the direction of the effect depended on the combination of nutrients added. Contrasts between the unamended control and each of the treatments (C, N, P, CN, NP, CNP) showed that all of the treatments containing P were greater than the control, treatments containing N alone or C + N were indistinguishable from the control, and the treatment containing only C was less than the control (Table 2).

**Table 2:** **Results of the nutrient amendment experiment showing directed comparisons between each treatment and the unamended control, based on all of the time points**.

	**Estimate (pmol leu l^−1^ h^−1^)**	**Standard error**	***t*-value**	***P*-value**	**95% Confidence limits**	
C	−0.03	0.008	−3.64	< 0.01	−4.4	−1.3
N	0.006	0.008	0.71	0.48	−1.0	2.1
P	0.03	0.008	3.92	< 0.001	1.5	4.7
CN	−0.003	0.008	−0.42	0.67	−1.9	1.2
NP	0.04	0.008	5.05	< 0.001	2.4	5.5
CNP	0.03	0.008	4.09	< 0.001	1.6	4.8

“*Estimate” is the estimated difference between the treatment and control. Negative estimates indicate control > treatments*.

**Figure 3:**
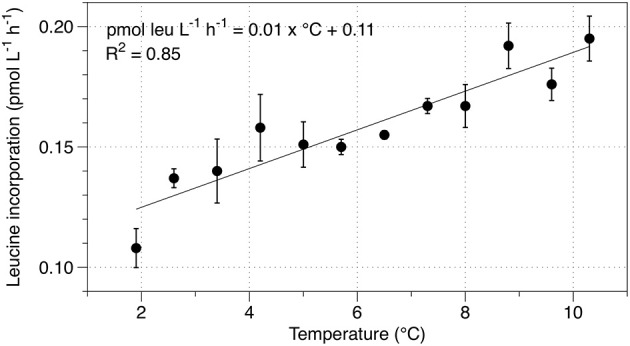
**Temperature response of leucine incorporation in the SLW water column**. Error bars indicate the standard error for three replicates.

To determine the heterotrophic response to temperature, samples amended with ^3^H-leucine were incubated over a gradient from 1.9 to 10.3°C. Rates of leucine incorporation increased by 0.01 pmol leu l^−1^ h^−1^ °C^−1^ of temperature increase (Figure 3). Based on the Arrhenius equation, the Q_10_ of the heterotrophic community is 2.13 and the activation energy for community leucine incorporation is 48.8 kJ mol^−1^.

### The SLW oxygen budget

When sampled, the water column was under-saturated with O_2_, relative to air-saturated water (~16% saturation; 71.9 μmol L^−1^; Christner et al., 2014). Rates of abiotic O_2_ consumption are unknown, but assumed to be minor compared to biological demand; therefore, biological activity was assumed as the only O_2_ sink. In order to, a) better understand the impact of biological activity on subglacial geochemistry, and b) determine the potential impact of subglacial hydrology (drain-fill cycles) on microorganisms in SLW, we constructed an O_2_ budget for the SLW water column, bounded in time by the year leading up to sampling. The budget is a simple mass balance of O_2_ sources (release in ice melt above the lake at 1 × 10^6^ m^3^ melt y^−1^, assuming an atmospheric concentration of O_2_ in the ice melt, and water inflow during the year prior to sampling (0.007 km^3^), assuming inflow concentration equal to that of SLW), and biological sinks in the water column and surface sediments (Table 3), and is based on the average concentration of dissolved O_2_ measured in SLW in 2013 (71.9 umol L^−1^). The model identified a major O_2_ sink as nitrification-driven chemoautotrophy in the water column (68% of total O_2_ demand). The remaining O_2_ demand was attributed to methane oxidation at the sediment-water interface (15% of total) and heterotrophic respiration in the water column and surface sediments (16% of the total O_2_ demand; 2.40 × 10^6^ mol O_2_ y^−1^). Assuming water starting at air saturation, the modeled consumption would produce the observed concentration of dissolved oxygen after ~40 years. The model also predicts that the SLW water column would become anoxic ~4 years after sampling, unless metabolic rates change, or a refilling event or elevation in the basal melt rate replenishes oxygen at a rate greater than our estimate.

**Table 3:** **Oxygen budget for SLW water column**.

	**Sources**			**Sinks**					**Annual O_2_ Deficit**	**Time until anoxia**	**Time since source**
	**Ice Melt**	**Inflow**	**Total Source**	**Water Auto**	**Water Hetero**	**Sediment Hetero**	**Sediment Methano**	**Total Sink**			
Moles O_2_ y^−1^	1.0 × 10^6^	5.0 × 10^5^	1.5 × 10^6^	2.6 × 10^6^	1.2 × 10^5^	4.9 × 10^5^	6.1 × 10^5^	3.9 × 10^6^	1.2 × 10^6^	4	39
% of total sink or source	67	33	–	68	3	13	16	–	–	–	–

*“Auto” refers to chemoautotrophy, “Hetero” refers to heterotrophy, “Methano” refers to methanotrophy. Sediment data are from the top 2 cm (Christner et al., 2014; Michaud, 2016). All “times” are in years. Annual oxygen deficit was computed by subtracting the total sinks from the sources. Time until anoxia was computed by dividing the measured oxygen concentration by the annual deficit. Time since source is the amount of time required to achieve the measured concentration of oxygen, starting at atmospheric equilibrium at standard temperature and pressure*.

### Energetics

**Figure 4:**
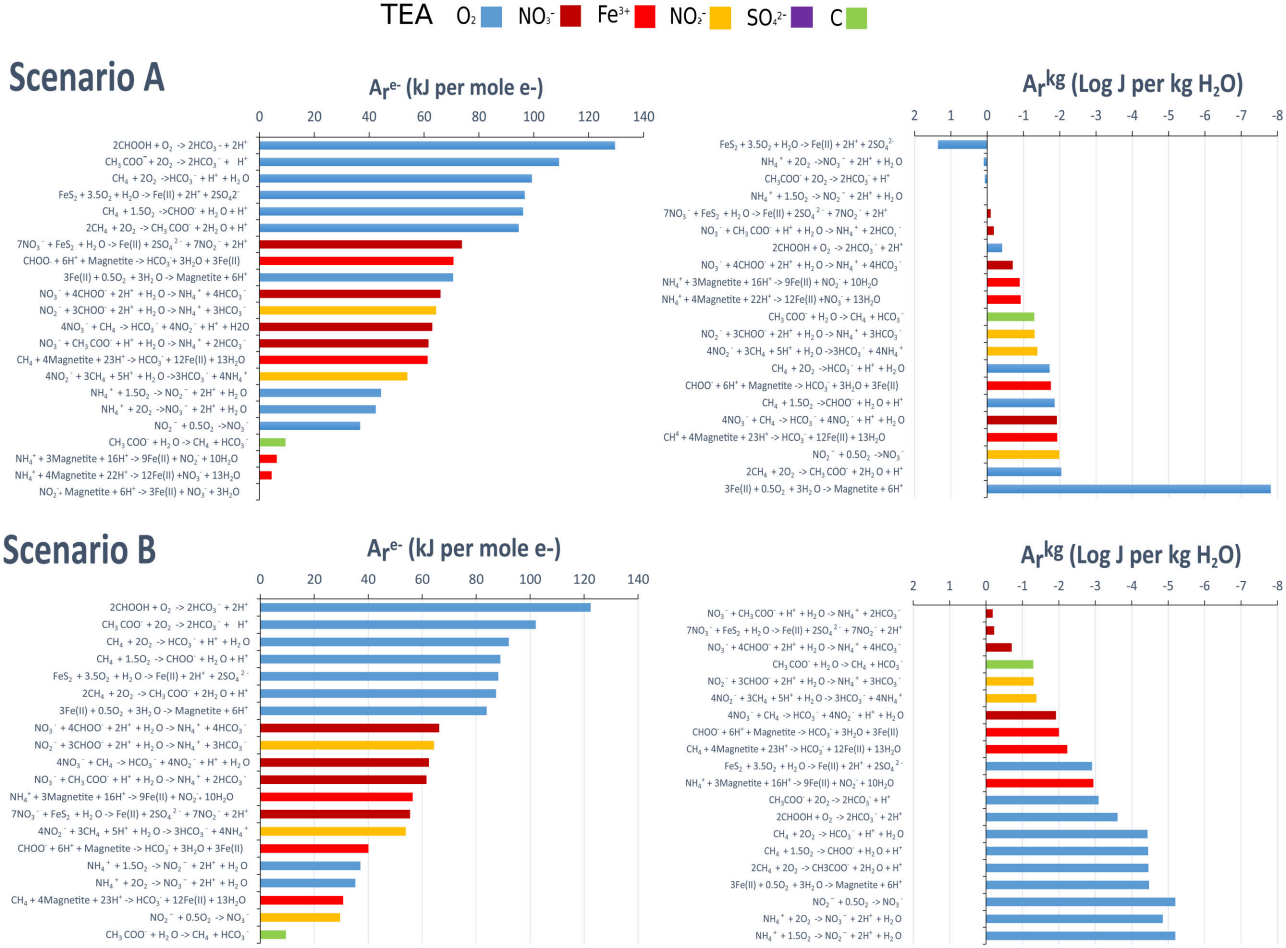
**Available chemical energy for potential metabolic reactions in the SLW water column**. Reaction choices were based upon the presence of reactants and products in SLW. The left hand panels show ranked (high to low) Gibbs energies in units of kJ per mole of electron transferred [A${}_{r}^{e-}$ (kJ per mole e^−^)]. The right hand panels show ranked (high to low) Gibbs energies of reaction as energy densities [A${}_{r}^{kg}$ (Log J per Kg H_2_O)]. Scenario A: Observed lake conditions (O_2_(aq) inclusive = 58 uM. pE = 6.45). Scenario B. Simulated midly anoxic. O_2_(aq) set at 1 nM. pE = 2. See Methods section for full details.

We used thermodynamic calculations to determine the energy available for metabolisms in the SLW water column under different lake redox scenarios (observed conditions and simulated anoxic conditions). The data are shown as kJ per mole of electron transferred (*A*${}_{r}^{e-}$) and as J per kg of H_2_O (*A*${}_{r}^{kg}$) (Figure 4; Table S2). Because we could not characterize or determine concentrations of all of the components of the dissolved organic carbon pool, our analyses focused on chemolithotrophic reactions, except for those involving the organic substrates methane, acetate, and formate, for which concentrations are known (Table S1).

Under oxic conditions (Scenario A) and modeled mildly anoxic conditions (Scenario B), energy yields (kJ mol^−1^ e^−^ transferred) were highest for the oxidation of acetate and formate, followed by the oxidation of methane. The chemolithotrophic metabolism with the highest energy yield was pyrite oxidation, with nitrifying metabolisms providing some of the lowest energy. In terms of energy density (J kg^−1^ H_2_O), pyrite oxidation was the most highly energetic in Scenario A, providing an order of magnitude higher energy yield than all other modeled metabolisms. Ammonia oxidation was the second most energetic metabolism, followed by acetate oxidation. In contrast with the calculations in terms of kJ per mole of electron transferred, methane oxidation was one of the least energetic metabolisms in terms of energy density. Under modeled mildly anoxic conditions (Scenario B), the dissimilatory reduction of nitrate to ammonia and nitrate-driven pyrite oxidation yielded the most energy.

## Discussion

SLW contains carbon and inorganic nutrient concentrations that are similar to those found in many freshwater and marine environments (Christner et al., 2014), yet the SLW microbial population was dominated by small, coccoid-shaped cells, similar to those found in low-energy environments (0.2–0.5 μm length; reviewed by (Lever et al., 2015); larger cells were also present, Mikucki et al., 2015). The low rates of heterotrophic activity relative to chemoautotrophic activity (Christner et al., 2014) and the corresponding low heterotrophic growth rates (Table 1) led us to examine the physiological ecology of microbial populations in the SLW water column, using a combination of experimental and modeling approaches.

### Energetics of heterotrophy in the SLW water column

In spite of high energy yields per mol e^−^ transferred associated with the oxidation of formate and acetate (highest of all reactions; *A*${}_{r}^{e-}$ = 129.6 and 109.2 kJ mol^−1^ e^−^, respectively) heterotrophic microorganisms in SLW had slow doubling times that were more similar to low-energy, subsurface environments than to surface environments (Table 4). When heterotrophic metabolisms were viewed in terms of energy density (*A*${}_{r}^{kg}$ = J kg^−1^ H_2_O), the energy yields were lower than both pyrite oxidation and ammonium oxidation. Given that energy density has been found to more closely reflect biomass than thermodynamic calculations normalized to mol e^−^ transferred (LaRowe and Amend, 2014), these results support our other data regarding low rates of heterotrophic activity in SLW.

**Table 4:** **Comparison between the physiological characteristics of heterotrophic microorganisms in SLW and those of other aquatic environments**.

**Location**	**Climate**	**Salinity**	**Doubling time (days)**	**Growth Efficiency**	**Leu:TdR**	**Reference**
Subglacial Lake Whillans	Polar (Antarctic)	Fresh	118	0.08	3.6	This study
Ross Sea	Polar (Antarctic)	Saline	4.33	0.24	9.2	Carlson et al., 1999; Ducklow et al., 2001
McMurdo Ice Shelf	Polar (Antarctic)	Saline	23.1	0.70	11	Vick-Majors, 2016
Northeast Atlantic Ocean	Subtropical	Saline	0.78 (0.46)	0.16 (0.15)	19.7 (12.6)	Alonso-Sáez et al., 2007
North Pacific Ocean	Temperate	Saline	0.33 (0.79)	0.15 (0.05)	8.9 (3.7)	del Giorgio et al., 2011
Svalbard (Kongsfjorden)	Polar (Arctic)	Saline	3.69	0.13	7.9	Motegi et al., 2013
East Lake Bonney	Polar (Antarctic)	Fresh	15.5 (39.1)	0.29 (0.05)^*^	18.6	Vick and Priscu, 2012
West Lake Bonney	Polar (Antarctic)	Fresh	7.2 (8.7)	0.32 (0.04)^*^	39.7	Vick and Priscu, 2012
Lake Fryxell	Polar (Antarctic)	Fresh	33.6 (77.9)	0.49 (0.07)^*^	59.1	Vick and Priscu, 2012
Mackenzie River	Polar (Arctic)	Fresh	1.79	0.65^*^	4.92	Galand et al., 2008
Beaufort Sea Coast	Polar (Arctic)	Saline	6.16	0.47^*^	5.50	Galand et al., 2008
Greenland	Polar (subarctic)	Saline	0.44	0.11^*^	69.1	Kaartokallio et al., 2013
Deep subsurface sediments	Temperate	Saline	~1–1000 years	–	–	Reviewed by Jørgensen (2011)

*Leu:Tdr is the molar ratio of leucine to thymidine incorporation. Doubling times were determined from published growth rates as ln2/growth rate, or from growth rates calculated from published rates of carbon production and bacterial abundance*.

* *indicates that published bacterial production data was used to estimate growth efficiency according to the relationship between bacterial respiration and bacterial production described by del Giorgio and Cole (1998) and used by Takacs et al. (2001) for Antarctic surface lakes. Parentheses indicate standard deviations calculated for studies that included multiple samples, either in space or time*.

The molar ratio of leucine incorporation to thymidine incorporation (Leu:TdR) in SLW was lower than most of the sites surveyed in our literature review, and was closest to those of riverine and coastal sites (Table 4). Leu:TdR has been inversely correlated to biomass production (Franco-Vidal and Morán, 2011), and used as an indicator of unbalanced growth (Chin-Leo and Kirchman, 1990). The discussion of relative changes in Leu:TdR, such as across seasons (e.g., Vick and Priscu, 2012) or along environmental gradients (e.g., Shiah and Ducklow, 1997), is most common, while the meaning of any individual ratio has not been quantified (Ducklow, 2000). The SLW water column Leu:TdR (3.6) was much higher than that of the SLW surface sediments (0.07; using the conversion factors in the Methods and the reported rates of leucine and thymidine incorporation for the top 2 cm of SLW sediments; 0.9 and 46.6 ng C d^−1^ gram dry weight sediment^−1^, respectively; Christner et al., 2014). It has been suggested that higher rates of leucine incorporation relative to thymidine incorporation may be a response to growth limiting conditions resulting in increased effort toward scavenging for substrate via the up-regulation of transport proteins (Church, 2008). SLW surface sediment concentrations of dissolved organic matter exceed those of the water column (Vick-Majors, 2016), implying that substrate availability may impact Leu:TdR in SLW. Similarly, if energy (in this case, organic carbon) is limiting, carbon is channeled to cellular maintenance functions rather than to cell division, corresponding to decreases in growth efficiency (del Giorgio et al., 2011).

The growth efficiency is defined as the percentage of biomass produced per unit of organic carbon consumed (del Giorgio and Cole, 1998) and is key for understanding the biogeochemistry of carbon in the environment (i.e., Carlson et al., 2007). Growth efficiency was low in the SLW water column compared to a number of surface environments, and was closest to that measured for a Svalbard fjord, and that estimated for a Greenland lake (Table 4). As a measure of the routing of carbon to catabolic or anabolic reactions, the low growth efficiency (8%) in SLW indicated that a large proportion of energy usage by SLW heterotrophic populations was for processes not related to anabolism (Carlson et al., 2007). This idea, coupled with the low (relative to chemolithotrophic metabolisms) energy density associated with heterotrophy (Figure 4), may help explain the apparently low growth rates (Table 1) in the SLW water column. The maintenance of non-growth related processes, which is often associated with low growth efficiency, may also function as an energetic trade-off, allowing cells to react to changing environmental conditions and take advantage of favorable circumstances for growth as they occur (del Giorgio and Cole, 1998).

The growth efficiency data show that heterotrophic microorganisms in SLW are channeling most of their carbon consumption (92% of the total carbon demand) to catabolic (represented here by heterotrophic respiration), rather than anabolic (heterotrophic production) processes. One possible explanation for this is that relict organic matter in SLW (Scherer et al., 1998; Wadham et al., 2012; Christner et al., 2014) may contain large molecules or colloids that must be broken down by extracellular enzymes before being utilized. The use of extracellular enzymes requires energy intensive production and excretion (Arnosti, 2011). Another, not necessarily exclusive, explanation is that the relative increase in catabolism reflects the maintenance of a range of cellular functions. The uncoupling of catabolism and anabolism is a microbial strategy for maintaining the metabolic flexibility that is key to responding to environmental changes (del Giorgio and Cole, 1998). The active hydrologic processes that are characteristic of SLW (cycles of filling and draining; Fricker et al., 2007; Siegfried et al., 2016) may lead to variations in lake physicochemical conditions, and could favor microorganisms that are able to respond to environmental changes. Similarly, some studies suggest that maintaining high concentrations of rRNA transcripts relative to rRNA genes also allows microorganisms to adapt to changing conditions (reviewed in Blazewicz et al., 2013). High ratios of amplified 16S rRNA transcripts to genes have been associated with certain taxa in SLW (Achberger et al., 2016). In SLW, low growth efficiency and high rRNA transcript-to-gene ratios suggest that heterotrophic microorganisms in the lake maintain metabolic flexibility that may be important in dealing with changing physicochemical conditions.

### Growth characteristics and the kinetics of leucine incorporation

Rates of leucine incorporation, a proxy for heterotrophic biomass production, were lower than V_max_ by a factor of ~3, in spite of the fact that the 20 nmol leu L^−1^ (20,000 pmol leu L^−1^) used in our incubations (Christner et al., 2014) appeared to be near the enzyme saturating concentration (Figure 1; asymptote of the fitted line indicates saturation). There are several possible explanations for this, which may each, in part, contribute to this discrepancy. Firstly, while the curve fit for our Michealis-Menten kinetics model had a low *P*-value (< 0.001), the fit only accounted for part of the variability in the data (*r*^2^ = 0.59). Secondly, because the ice above the lake was flowing across the lake throughout our sampling period (Tulaczyk et al., 2014), the cast 2 sample point (when kinetic experiments were conducted) may have encountered different lake conditions. Differences related to our movement across the lake surface, while not detected explicitly in our data set, may have impacted the activity of the microorganisms in cast 2. The fact that the rate of leucine incorporation determined for cast 2 was closer to V_max_ than those of the other two casts (2x lower than V_max_ for cast 2 vs. 3x lower for cast 1 and cast 3) suggests that perhaps this latter explanation is important. No significant differences in microbial community structure were detected between casts (Christner et al., 2014), so our results do represent the microbial community present in SLW during the sampling period.

The K_t_ value for leucine incorporation in SLW (5459 pmol L^−1^) was substantially lower than values published for other systems (e.g., oligotrophic ocean waters, ~2 × 10^4^ pmol L^−1^, Williams and Hobbie, 2012; eutrophic hard water lake, ~3 × 10^4^ pmol L^−1^, Buesing and Gessner, 2003). A lower value of K_t_ should be associated with higher affinity incorporation systems, and adaptation to lower concentrations of natural substrate (Wright and Hobbie, 1966). These data suggest that, if leucine is a proxy for the incorporation of organic C (Calvo-Díaz and Morán, 2009), heterotrophic populations in SLW should be able to take up C at low concentrations. This, combined with the relatively high concentration of DOC in the SLW water column (221 μmol L^−1^) and the presence of labile carbon substrates (acetate and formate), suggest that heterotrophic growth is not expected to be limited by the bulk substrate concentration.

Factors in addition to substrate quantity can limit heterotrophic metabolism (catabolism, anabolism, or both), including substrate quality (Jagadamma et al., 2014), mode and timing of substrate production (Carlson, 2002; Jiao et al., 2010), temperature (He et al., 2014), nutrient availability (Morita, 1997), and, in terms of the energy available from metabolic reactions, the redox environment (Brewer et al., 2014). We discuss these below.

### Potential limitations on heterotrophic activity

The substrate released by chemosynthetic and heterotrophic microorganisms may be of lower quality than freshly-produced photosynthate available in surface environments (Ogawa et al., 2001). While rates of chemosynthesis exceed rates of heterotrophic carbon incorporation and respiration by a factor of 1.5 in SLW (Christner et al., 2014; Vick-Majors, 2016), the bulk of the chemosynthetically-fixed carbon may be retained by chemosynthetic cells and therefore not be immediately available to support heterotrophic growth (Kawasaki and Benner, 2006; Kawasaki et al., 2013), leading to an offset between the heterotrophic demand for carbon and its supply (Carlson, 2002). Similarly, evaluations of the quality of dissolved organic matter in SLW, based on the ratio of dissolved organic C to dissolved organic N (molar DOC:DON ~95; Vick-Majors, 2016) suggest low overall lability of the bulk pool of dissolved organic matter (Hunt et al., 2000; Wiegner and Seitzinger, 2004). Our energetic calculations show that the concentrations of acetate and formate, the labile carbon substrates we measured, may limit the energetic favorability of some heterotrophic metabolisms (Table S2).

Additions of inorganic macronutrients (N and P) had a small positive effect on heterotrophic biomass production. The response to P was contrary to limiting nutrients predicted by lake water geochemistry. Similar to other subglacial environments (Boyd et al., 2011), SLW had low concentrations of nitrogen relative to carbon (molar ratio of dissolved organic C to dissolved inorganic *N* = 66.9 (Christner et al., 2014). The dissolved organic N (Vick-Majors, 2016) is a substantial part of the dissolved N pool (organic C:total N molar ratio 39.6), but its inclusion still yields values higher than the ideal C:N ratio of 6.6 (Redfield et al., 1963). The molar ratio of dissolved inorganic N to dissolved inorganic P in SLW (1.1; Christner et al., 2014) was lower than the Redfield ratio (N:P = 16), further supporting N-deficiency. One possible explanation for the apparent disagreement between the lake geochemistry and the biological response to nutrient amendment is that the waters in SLW are oversaturated with respect to apatite minerals (Saturation Index FCO_3_Apatite = 15.27; Hydroxylapatite = 3.52 in SLW water column from Thermodynamic Scenario A; data not shown). Such minerals may not be immediately bioavailable but, given the small size of suspended particulate matter in the SLW water column (Tulaczyk et al., 2014), may have passed through the GFF filters used for sample processing and been dissolved during P analysis by the molybdenum blue method (Strickland and Parsons, 1968), leading to overestimation of dissolved inorganic P. Arsenate, whose concentration is unknown in the SLW water column, can also interfere with the determination of P, and acid-labile dissolved organic P may also be detected, resulting in a relative inflation of the size of the dissolved inorganic P pool (see, for example, Nagul et al., 2015). Interestingly, the addition of glucose consistently had a negative effect on heterotrophic activity. We do not have a simple biochemical explanation for this, except to hypothesize that is not a preferred carbon source for organisms in the SLW water column.

Heterotrophic carbon production increased with experimental temperature increases, as predicted. A linear fit described 85% of the variation in the dataset (Figure 3), which may imply that different heterotrophic groups responded differently to the changes in temperature, but we were unable to test this using our bulk experiments. The Q_10_ value of 2.13 determined in our experiments is similar to that commonly observed in biological systems (typically ~2–3) and to the empirically determined Q_10_ for heterotrophic production in the outflow from an Antarctic subglacial environment in the McMurdo Dry Valleys (Blood Falls, Q_10_ = 1.74; Mikucki et al., 2004). The activation energy we determined for heterotrophic carbon production in SLW (48.8 kJ mol^−1^) is higher than the calculated global ocean average of 11 kJ mol^−1^, but lower than the average determined from experiments similar to ours on samples of marine water (64 kJ mol^−1^; Kirchman et al., 2009). Together, these data comparisons imply that the heterotrophic microbial community in SLW is no more sensitive to or limited by temperature than other aquatic microbial communities. Therefore, factors other than temperature should contribute to limitation of heterotrophic activity in SLW.

### Caveats on and conclusions from thymidine and leucine incorporation experiments

Together, the experimental data on heterotrophic activity in SLW show that the populations were growing slowly compared to a range of surface environments (Table 4), and that their growth was inefficient, in spite of the presence of energetic substrates (acetate and formate) and adaptation to low substrate concentrations (based on leucine-derived K_t_). It should be noted that our growth rate and doubling time calculations assume that all cells are active and capable of incorporating leucine or thymidine. This is a common method for estimating heterotrophic microbial growth rates (e.g., Straza et al., 2010), but means that our growth rates are minimum estimates, while our doubling times are maximum estimates. A small number of more rapidly growing cells may be responsible for the heterotrophic activity we detected in the water column. Our kinetic experiments showed that the rates of leucine incorporation that we measured were < V_max_, in spite of apparently near saturating concentrations of substrate in our incubations. The incorporation rates determined during cast 2, when kinetic experiments were conducted, were also higher than those of the other casts. Thus, unaccounted for differences in lake or incubation conditions may have led to an overestimate of V_max_. If leucine incorporation was not saturated, and thymidine incorporation was, then this could also impact the Leu:TdR ratio. There can also be variations among taxa in their ability to incorporate leucine and thymidine (Pérez et al., 2010), which can impact the Leu:TdR ratio. Based on our experimental data, the availability of inorganic nutrients, the timing of organic C substrate availability, and/or the availability of high quality organic C substrates appear to be the most likely variables limiting heterotrophic growth.

### The oxygen budget and microbial energetics

Despite low growth rates, aerobic heterotrophic and chemoautotrophic metabolisms in SLW imposed a significant oxygen demand on the water column. Because the source water originates from glacial melt, which we parameterize as containing atmospheric concentrations of oxygen, and the lake is not ventilated, our model can provide an estimate for the amount of time elapsed since the water in SLW was frozen in the overlying ice, assuming constant O_2_ consumption rates. We can also use the model to examine how microbial physiology in the lake could drive it from an oxidizing to a reducing environment in the absence of increased inputs of oxygenated water.

Based on the oxygen required to support the rates of heterotrophic incorporation and respiration of organic C in the SLW surface sediments and water column (Christner et al., 2014; Vick-Majors, 2016), modeled methane oxidation rates for the surface sediments (Michaud, 2016), and chemoautotrophic activity supported by nitrification in the water column, our model shows that it would take ~40 years for air-saturated water in SLW to reach the observed oxygen concentration. The model results also show that the SLW water column would become anoxic ~4 years after it was sampled, assuming freshly melted, oxygen-containing water enters the basin at a rate of 0.007 km^3^ y^−1^ (inflow rate during the year prior to sampling; Siegfried et al., 2016). Mikucki et al. (2015) determined rates of chemoautotrophic production for the sediment-water interface, which exceeded those determined for the water column. High rates of chemoautotrophic activity at the sediment-water interface could deplete oxygen more quickly, but we were not able to estimate rates for this process. Replacing the nitrification-based chemoautotrophic oxygen demand with a pyrite oxidation-based demand (highest calculated chemolithotrophic energy yield; Figure 4) and using the stoichiometry of pyrite-oxidation mediated carbon fixation determined for another subglacial environment (range 7–24 mol SO${}_{4}^{2-}$ produced per mol C fixed; Boyd et al., 2014), results in a similar model prediction for the depletion of oxygen (~2–7 years). This phenomenon of “chemical switching” between oxic and anoxic conditions has been described under conditions of delayed flow of subglacial waters from Arctic mountain glaciers (Wynn et al., 2006), but we present its first application to the understanding of subglacial microbiological and hydrological processes under the Antarctic ice sheet.

Given the relatively short predicted time scales for oxygen depletion, SLW and the interconnected hydrological network in this region may undergo cycles of anoxia based on subglacial hydrology and biological activity, but the following caveats should be considered. Fill/drain cycles on the order of years have been observed during the last ~10 years of observations for SLW and other lakes in the region (Siegfried et al., 2016). The average fill rate during the period for which data are available (2008–2014) was 0.009 km^3^ y^−1^ (calculated from data in Siegfried et al., 2016), suggesting that our model, which used 0.007 km^3^ y^−1^, is a good representation of this time period. According to our model results, the SLW water column was trending toward anoxia at the time of sampling, and would continue to do so until (presumably) oxygenated water was supplied from upstream. Indeed, a fast filling event was detected beginning in 2014, immediately after we sampled the lake (Siegfried et al., 2016). Whether the inflow-supplied water was oxygenated or not is unknown, but if oxygen is at steady-state underneath the ice, upstream water should contain the same oxygen concentration as SLW. In this case, we estimate that the fast filling event would have provided ~10^8^ moles of oxygen to SLW during the year following our sample collection, and maintained the oxic water column. The oxygen concentrations measured in SLW could also be maintained in spite of fill/drain cycles if the microbial oxygen demand estimated in our model is too high, if the estimated inputs of oxygen are too low, or if the basal melt rate were to become elevated. The basal melt rate is determined by the geothermal heat flux (Fisher et al., 2015), making an elevation in melt rate unlikely. A complete understanding of the oxygen dynamics in SLW, and other subglacial lakes, will require further geochemical, hydrological, and biological observations.

Nitrification has been shown to be an important process in other subglacial environments, even though these environments are often nitrogen limited (Boyd et al., 2011). Because the rate of biological oxygen drawdown in our model is highly dependent on estimated rates of nitrification in the SLW water column (68% of oxygen demand), and the ammonium-dominated N-pool is stoichiometrically limiting in the SLW water column (Christner et al., 2014) we discuss the balance between the dynamics of oxygen and ammonium in more detail.

Ammonium can be added to aquatic environments via N-fixation, atmospheric deposition of ammonia (mainly agriculturally sourced), and/or run-off from agriculturally-impacted soils. SLW is relatively isolated from anthropogenic inputs, and a metagenomic analysis of the microbial community does not indicate that N-fixation as an important process in the lake (Achberger, 2016). The nitrate in SLW is microbially, rather than atmospherically, derived (Christner et al., 2014), implying that nitrification and biological recycling of organic N in the water column and/or sediment porewaters, are the major sources of N to the water column. The major sources of ammonium to the SLW basin are therefore hypothesized to be upward diffusion of relict ammonium from the sediment porewaters and the inflow of ammonium-containing waters from upstream.

The SLW sediment porewaters are ammonium-rich relative to the water column and provide a flux of 8.2 × 10^2^ mol NH${}_{4}^{+}$ y^−1^ to the water column (Vick-Majors, 2016). If we make the same assumptions as in our oxygen model (water inflow rate = 0.007 km^3^ y^−1^; upstream water is geochemically identical to the water in SLW with NH${}_{4}^{+}$ = 3.3 μmol L^−1^; Table S1), then inflow from upstream adds 2.3 × 10^4^ mol NH${}_{4}^{+}$ y^−1^, for a total annual ammonium supply of 2.4 × 10^4^ mol NH${}_{4}^{+}$ y^−1^. The SLW water column ammonium demand is estimated to be as high as 1.3 × 10^6^ mol NH${}_{4}^{+}$ y^−1^, assuming all chemoautotrophic C-fixation is due to nitrification (2.7 nmol C L^−1^ d^−1^; Christner et al., 2014) and that the stoichiometry associated with chemoautotrophic nitrification is 10 mol N per mol of C fixed; Berg et al., 2015). Subtracting the demand (1.3 × 10^6^ mol NH${}_{4}^{+}$ y^−1^) from the supply (2.4 × 10^4^ mol NH${}_{4}^{+}$ y^−1^) leaves an annual ammonium deficit of 1.3 × 10^6^ mol NH${}_{4}^{+}$ y^−1^. At this rate, our model predicts that the SLW ammonium pool (4.4 × 10^5^ mol NH${}_{4}^{+}$; based on the SLW ammonium concentration in Table S1 and a 0.132 km^3^ lake volume—see Methods) would be depleted in < 1 year. This is shorter than the ~4 year depletion time modeled for oxygen in the SLW water column. It is clear that if nitrification is an important process in SLW, as geochemical, isotopic, and molecular data suggest (Christner et al., 2014; Achberger, 2016; Achberger et al., 2016), then unaccounted for sources (such as overlying ice) and the internal cycling of N also play important roles.

We used the results of thermodynamic calculations to further investigate modes of N-cycling in the SLW water column. Ammonium oxidation and nitrite oxidation are energetically feasible under the conditions observed in SLW, and high rRNA:rDNA ratios for nitrite oxidizing bacteria suggest that they are active in the SLW water column (Achberger et al., 2016). Our energy density calculations suggest that ammonium oxidation is the second most energetic metabolism in the SLW water column, which is consistent with predictions of its importance in N-cycling and primary production in SLW (Christner et al., 2014). Ammonium can be regenerated from biomass mineralization, from the dissolved organic N pool, and via dissimilatory nitrate reduction to ammonium (DNRA), an anaerobic process that couples the reduction of nitrate to the oxidation of sulfide or organic carbon (Giblin et al., 2013). DNRA coupled to organic carbon oxidation was the most energetic metabolism in terms of energy density in the simulated anoxic SLW (Figure 4). Under anoxic conditions, DNRA must compete with denitrification, which converts NO${}_{3}^{-}$ to gaseous intermediates and end products (N_2_O, N_2_). As such, denitrification results in the loss of N (in the absence of N-fixation), while DNRA provides a readily available source of inorganic fixed N. Metagenomic data from SLW (Achberger, 2016) shows the genetic potential for denitrification in the lake, but we were not able to measure N_2_, preventing efforts to calculate the energetics of denitrification. DNRA is less oxygen sensitive than denitrification (Fazzolari et al., 1998), is favored when organic carbon concentrations are high (Giblin et al., 2013), and may be favored at high C:N ratios (Tiedje et al., 1983) such as those in SLW (Christner et al., 2014); therefore, it may successfully compete with denitrification in this system. Periodic changes in lake redox resulting from biological oxygen consumption may be important for the regeneration of the ammonium pool in SLW, and allow for the continuation of metabolic activity sustained by relict N stored in the sediments.

## Conclusions

Subglacial Lake Whillans maintains an active, but slow growing population of heterotrophic microorganisms that are ultimately dependent on relict organic matter and microbially produced organic matter. The heterotrophic community in SLW appears to be limited by the energy available from the oxidation of organic matter in the oxygenated water column, and by the availability of inorganic nutrients. The oxidation of methane sourced from deeper lake sediments (Wadham et al., 2012), reduced minerals (e.g., pyrite, ammonium), and labile organics support respiration rates that exceed the estimated supply of oxygen to the SLW water column. This suggests the occurrence of dynamic shifts in redox states which are directly linked to both subglacial hydrology and microbial community metabolism. Our data, together with molecular evidence for the presence of organisms adapted to a range of redox conditions (Achberger et al., 2016) suggest that the SLW microbial community is physiologically adapted to fluctuating environmental conditions. The redox changes that we predict may, in part, drive biogeochemical cycles beneath the ice, allowing for the regeneration of nitrogen compounds in this stoichiometrically N-limited environment. The implications of redox shifts for microbial diversity, energetics, and biogeochemical cycling beneath the ice sheet warrants more detailed investigation.

## Author contributions

TV wrote the manuscript, collected samples, designed/performed experiments, contributed to the oxygen budget, and interpreted the data; ACM performed field experiments, performed thermodynamic calculations, wrote the thermodynamic calculation methods, generated thermodynamics table, and assisted in data interpretation; AA assisted with field experiments and sample collection, provided interpretation of data and comparison to molecular data; BC collected samples and provided interpretation of data and comparison to molecular data; JD contributed to the oxygen budget and assisted with data interpretation; ABM assisted with field experiments and sample collection and contributed to the oxygen budget; JM assisted with field experiments, sample collection, and data interpretation; AP assisted with data interpretation and comparisons to molecular data; MS assisted with field experiments, sample collection, and geochemical data interpretation; JP designed experiments, collected samples, assisted with sample processing, and contributed to data interpretation. All authors contributed to the writing of the manuscript.

## Funding

The Whillans Ice Stream Subglacial Access Research Drilling (WISSARD) project was funded by National Science Foundation grants (0838933, 0838896, 0838941, 0839142, 0839059, 0838885, 0838763, 0839107, 0838947, 0838854, 0838764, and 1142123) from the Division of Polar Programs. Partial support was also provided by funds from NSF award 1023233 (BC), NSF award 1115245 (JP), the NSF's Graduate Research Fellowship Program (1247192; AA), and fellowships from the American Association of University Women (TV) and NSF's IGERT Program (0654336) and NSF's Center for Dark Energy Biosphere Investigations (AM), and a Sêr Cymru National Research Network for Low Carbon, Energy and the Environment grant from the Welsh Government and Higher Education Funding Council for Wales (AM).

### Conflict of interest statement

The authors declare that the research was conducted in the absence of any commercial or financial relationships that could be construed as a potential conflict of interest.
