# Supplementary material for: Physiological Ecology of Microorganisms in Subglacial Lake Whillans
Source: Front Microbiol. 2016 Oct 27;7:1705. doi: 10.3389/fmicb.2016.01705 (PMC5081474; doi:10.3389/fmicb.2016.01705)
Supplement: Supplementary file 1 [file Table1.PDF]

Table S1. Measured SLW water chemistry data. Values from Cast 1 were used in thermodynamic calculations. *mg L^{-1}, **=μmol L^{-1}, ***mmol L^{-1}. Cast 1, 2, and 3 refer to the three individual hydrocasts in the SLW water column. '•'Temperature was determined from CTD casts, which were carried out separately from the hydrocasts. '-' no data.

Parameter	***Cast 1***	***Cast 2***	***Cast 3***	***Average (+/- s.d.)***	***Reference***
Temperature (^{o}C)	•	•	•	-0.5 (0.03)	Christner et al., 2014
pH	8.0	8.2	8.2	8.1 (0.10)	Christner et al., 2014
Redox (mV [SHE])/pE	380/6.45	-	-	-	Christner et al., 2014
Dissolved oxygen*	2.0	2.6	2.3	2.3 (0.4)	Christner et al., 2014
Acetate**	1.5	1.3	1.1	1.3 (0.20)	Christner et al., 2014
Formate**	1.7	1.0	1.0	1.2 (0.30)	Christner et al., 2014
DIC***	2.09	2.13	2.11	2.11 (0.02)	Christner et al., 2014
CH_4^{**}	0.02	-	-	-	Michaud, et al., in preparation
NO_3^{-**}	1.3	0.96	0.42	0.82 (0.47)	Christner et al., 2014
NO_2^{-**}	0.04	0.05	0.17	0.09 (0.07)	Christner et al., 2014
NH_4^{+**}	2.9	2.6	1.7	2.4 (0.63)	Christner et al., 2014
PO_4^{3-**}	3.1	2.5	3.8	3.1 (0.7)	Christner et al., 2014
SO_4^{2-**}	549	559	559	556 (5.8)	Christner et al., 2014
Dissolved Fe**	0.006	-	-	-	Turetta, et al., in preparation
