# Supplementary material for: Physiological Ecology of Microorganisms in Subglacial Lake Whillans
Source: Front Microbiol. 2016 Oct 27;7:1705. doi: 10.3389/fmicb.2016.01705 (PMC5081474; doi:10.3389/fmicb.2016.01705)
Supplement: Supplementary file 2 [file Table2.pdf]

Table S2. Available chemical energy for potential metabolic reactions in SLW. Reaction choice based upon the presence of aqueous reactants and products in SLW. (A) shows ranked (high to low) Gibbs energies in units of kJ per mole of electron transferred [$A_r^{e-}$ (kJ per mole $e^-$)]. (B) shows ranked (high to low) Gibbs energies of reaction as energy densities [$A_r^{kg}$ (Log J per Kg $H_2O$)]. Scenario A: Observed lake conditions ($O_2$(aq) inclusive = 58uM. pE = 6.45). Scenario B. Simulated mildly anoxic. $O_2$(aq) set at 1 nM. pE = 2. SLW data are from cast 1. See methods section for full details.

(A)

| **Scenario A. Observed SLW. $O_2$ inclusive 58uM. pE = 6.45.** | **TEA** | **# e-** | **$A_r^{e-}$ (kJ per mole e-)** | **$A_r^{kg}$ (J per Kg $H_2O$)** |
|---|---|---|---|---|
| $2CHOOH + O_2 \rightarrow 2HCO_3^- + 2H^+$ | $O_2$ | 4 | 129.6 | $3.86 \times 10^{-1}$ |
| $CH_3COO- + 2O_2 \rightarrow 2HCO_3^- + H^+$ | $O_2$ | 8 | 109.2 | 1.16 |
| $CH_4 + 2O_2 \rightarrow HCO_3^- + H^+ + H_2O$ | $O_2$ | 8 | 99.3 | $1.91 \times 10^{-2}$ |
| Pyrite + $3.5O_2 + H_2O \rightarrow Fe^{2+} + 2H^+ + 2SO_4^{2-}$ | $O_2$ | 14 | 96.7 | 22.7 |
| $CH_4 + 1.5O_2 \rightarrow CHOO^- + H_2O + H^+$ | $O_2$ | 6 | 96.1 | $1.38 \times 10^{-2}$ |
| $2CH_4 + 2O_2 \rightarrow CH_3COO^- + 2H_2O + H^+$ | $O_2$ | 8 | 94.5 | $9.07 \times 10^{-3}$ |
| $7NO_3^-$ + Pyrite + $H_2O \rightarrow Fe^{2+} + 2SO_4^{2-} + 7NO_2^- + 2H^+$ | $NO_3^-$ | 14 | 73.8 | $7.97 \times 10^{-1}$ |
| $CHOO^- + 6H^+$ + Magnetite $\rightarrow HCO_3^- + 3H_2O + 3Fe^{2+}$ | $Fe^{3+}$ | 2 | 70.7 | $1.76 \times 10^{-2}$ |
| $3Fe^{2+} + 0.5O_2 + 3H_2O \rightarrow$ Magnetite + $6H^+$ | $O_2$ | 2 | 70.6 | $1.54 \times 10^{-8}$ |
| $NO_3^- + 4CHOO^- + 2H^+ + H_2O \rightarrow NH_4^+ + 4HCO_3^-$ | $NO_3^-$ | 8 | 66.0 | $1.97 \times 10^{-1}$ |
| $NO_2^- + 3CHOO^- + 2H^+ + H_2O \rightarrow NH_4^+ + 3HCO_3^-$ | $NO_2^-$ | 6 | 64.4 | $4.95 \times 10^{-2}$ |
| $4NO_3^- + CH_4 \rightarrow HCO_3^- + 4NO_2^- + H^+ + H_2O$ | $NO_3^-$ | 8 | 63.0 | $1.20 \times 10^{-2}$ |
| $NO_3^- + CH_3COO^- + H^+ + H_2O \rightarrow NH_4^+ + 2HCO_3^-$ | $NO_3^-$ | 8 | 61.6 | $6.55 \times 10^{-1}$ |
| $CH_4$ + 4Magnetite + $23H^+ \rightarrow HCO_3^- + 12Fe^{2+} + 13H_2O$ | $Fe^{3+}$ | 8 | 61.3 | $1.18 \times 10^{-2}$ |
| $4NO_2^- + 3CH_4 + 5H^+ + H_2O \rightarrow 3HCO_3^- + 4NH_4^+$ | $NO_2^-$ | 24 | 53.9 | $4.14 \times 10^{-2}$ |
| $NH_4^+ + 1.5O_2 \rightarrow NO_2^- + 2H^+ + H_2O$ | $O_2$ | 6 | 44.3 | $9.74 \times 10^{-1}$ |
| $NH_4^+ + 2O_2 \rightarrow NO_3^- + 2H^+ + H_2O$ | $O_2$ | 8 | 42.4 | 1.24 |
| $NO_2^- + 0.5O_2 \rightarrow NO_3^-$ | $O_2$ | 2 | 36.7 | $1.03 \times 10^{-2}$ |
| $CH_3COO^- + H_2O \rightarrow CH_4 + HCO_3^-$ | C | 4 | 9.5 | $5.03 \times 10^{-2}$ |
| $NH_4^+$ + 3Magnetite + $16H^+ \rightarrow 9Fe^{2+} + NO_2^- + 10H_2O$ | $Fe^{3+}$ | 6 | 6.3 | $1.27 \times 10^{-1}$ |
| $NH_4^+$ + 4Magnetite + $22H^+ \rightarrow 12Fe^{2+} + NO_3^- + 13H_2O$ | $Fe^{3+}$ | 8 | 4.5 | $1.19 \times 10^{-1}$ |
| $NO_2^-$ + Magnetite + $6H^+ \rightarrow 3Fe^{2+} + NO_3^- + 3H_2O$ | $Fe^{3+}$ | 2 | -1.2 | $-3.02 \times 10^{-4}$ |
| **Scenario B. Simulated anoxic. O2 set at 1 nM. pE = 2** | **TEA** | **# e-** | **$A_r^{e-}$ (kJ per mole e-)** | **$A_r^{kg}$ (J per Kg $H_2O$)** |
| $2CHOOH + O_2 \rightarrow 2HCO_3^- + 2H^+$ | $O_2$ | 4 | 122.4 | $2.45 \times 10^{-4}$ |
| $CH_3COO^- + 2O_2 \rightarrow 2HCO_3^- + H^+$ | $O_2$ | 8 | 102.1 | $8.16 \times 10^{-4}$ |
| $CH_4 + 2O_2 \rightarrow HCO_3^- + H^+ + H_2O$ | $O_2$ | 8 | 92.1 | $3.68 \times 10^{-5}$ |
| $CH_4 + 1.5O_2 \rightarrow CHOO^- + H_2O + H^+$ | $O_2$ | 6 | 89.0 | $3.56 \times 10^{-5}$ |
| Pyrite + $3.5O_2 + H_2O \rightarrow Fe^{2+} + 2H^+ + 2SO_4^{2-}$ | $O_2$ | 14 | 88.2 | $1.23 \times 10^{-3}$ |
| $2CH_4 + 2O_2 \rightarrow CH_3COO^- + 2H_2O + H^+$ | $O_2$ | 8 | 87.4 | $3.49 \times 10^{-5}$ |
| $3Fe^{2+} + 0.5O_2 + 3H_2O \rightarrow$ Magnetite + $6H^+$ | $O_2$ | 2 | 83.9 | $3.36 \times 10^{-5}$ |
| $NO_3^- + 4CHOO^- + 2H^+ + H_2O \rightarrow NH_4^+ + 4HCO_3^-$ | $NO_3^-$ | 8 | 66.3 | $1.97 \times 10^{-1}$ |
| $NO_2^- + 3CHOO^- + 2H^+ + H_2O \rightarrow NH_4^+ + 3HCO_3^-$ | $NO_2^-$ | 6 | 64.4 | $4.95 \times 10^{-2}$ |
| $4NO_3^- + CH_4 \rightarrow HCO_3^- + 4NO_2^- + H^+ + H_2O$ | $NO_3^-$ | 8 | 62.5 | $1.20 \times 10^{-2}$ |
| $NO_3^- + CH_3COO^- + H^+ + H_2O \rightarrow NH_4^+ + 2HCO_3^-$ | $NO_3^-$ | 8 | 61.6 | $6.55 \times 10^{-1}$ |
| $NH_4^+$ + 3Magnetite + $16H^+ \rightarrow 9Fe^{2+} + NO_2^- + 10H_2O$ | $Fe^{3+}$ | 6 | 56.5 | $1.13 \times 10^{-3}$ |
| $7NO_3^-$ + Pyrite + $H_2O \rightarrow Fe^{2+} + 2SO_4^{2-} + 7NO_2^- + 2H^+$ | $NO_3^-$ | 14 | 55.5 | $5.99 \times 10^{-1}$ |
| $4NO_2^- + 3CH_4 + 5H^+ + H_2O \rightarrow 3HCO_3^- + 4NH_4^+$ | $NO_2^-$ | 24 | 53.9 | $4.14 \times 10^{-2}$ |
| $CHOO^- + 6H^+$ + Magnetite $\rightarrow HCO_3^- + 3H_2O + 3Fe^{2+}$ | $Fe^{3+}$ | 2 | 40.1 | $9.96 \times 10^{-3}$ |
| $NH_4^+ + 1.5O_2 \rightarrow NO_2^- + 2H^+ + H_2O$ | $O_2$ | 6 | 37.1 | $6.36 \times 10^{-6}$ |
| $NH_4^+ + 2O_2 \rightarrow NO_3^- + 2H^+ + H_2O$ | $O_2$ | 8 | 35.2 | $1.41 \times 10^{-5}$ |
| $CH_4$ + 4Magnetite + $23H^+ \rightarrow HCO_3^- + 12Fe^{2+} + 13H_2O$ | $Fe^{3+}$ | 8 | 30.7 | $5.90 \times 10^{-3}$ |
| $NO_2^- + 0.5O_2 \rightarrow NO_3^-$ | $O_2$ | 2 | 29.6 | $6.36 \times 10^{-6}$ |
| $CH3COO^- + H_2O \rightarrow CH_4 + HCO_3^-$ | C | 4 | 9.5 | $5.03 \times 10^{-2}$ |
| $NH_4^+$ + 3Magnetite + $16H^+ \rightarrow 9Fe^{2+} + NO_2^- + 10H_2O$ | $Fe^{3+}$ | 6 | -24.3 | $-4.87 \times 10^{-1}$ |
| $NH_4^+$ + 4Magnetite + $22H^+ \rightarrow 12Fe^{2+} + NO_3^- + 13H_2O$ | $Fe^{3+}$ | 8 | -26.2 | $-6.99 \times 10^{-1}$ |
| $NO_2^-$ + Magnetite + $6H^+ \rightarrow 3Fe^{2+} + NO_3^- + 3H_2O$ | $Fe^{3+}$ | 2 | -31.8 | $-8.14 \times 10^{-3}$ |

(B)

| **Scenario A. Observed SLW. $O_2$ inclusive 58uM. pE = 6.45.** | **TEA** | **# e-** | **$A_r^{e-}$ (kJ per mole e-)** | **$A_r^{kg}$ (J per Kg $H_2O$)** |
|---|---|---|---|---|
| Pyrite + $3.5O_2$ + $H_2O$ → $Fe^{2+}$ + $2H^+$ + $2SO_4^{2-}$ | $O_2$ | 14 | 96.7 | 22.7 |
| $NH_4^+$ + $2O_2$ → $NO_3^-$ + $2H^+$ + $H_2O$ | $O_2$ | 8 | 42.4 | 1.24 |
| CH3COO$^-$ + $2O_2$ → $2HCO_3^-$ + $H^+$ | $O_2$ | 8 | 109.2 | 1.16 |
| $NH_4^+$ + $1.5O_2$ → $NO_2^-$ + $2H^+$ + $H_2O$ | $O_2$ | 6 | 44.3 | $9.74 \times 10^{-1}$ |
| $7NO_3^-$ + Pyrite + $H_2O$ → $Fe^{2+}$ + $2SO_4^{2-}$ + $7NO_2^-$ + $2H^+$ | $NO_3^-$ | 14 | 73.8 | $7.97 \times 10^{-1}$ |
| $NO_3^-$ + $CH_3COO^-$ + $H^+$ + $H_2O$ → $NH_4^+$ + $2HCO_3^-$ | $NO_3^-$ | 8 | 61.6 | $6.55 \times 10^{-1}$ |
| 2CHOOH + $O_2$ → $2HCO_3^-$ + $2H^+$ | $O_2$ | 4 | 129.6 | $3.86 \times 10^{-1}$ |
| $NO_3^-$ + $4CHOO^-$ + $2H^+$ + $H_2O$ → $NH_4^+$ + $4HCO_3^-$ | $NO_3^-$ | 8 | 66.0 | $1.97 \times 10^{-1}$ |
| $NH_4^+$ + 3Magnetite + $16H^+$ → $9Fe^{2+}$ + $NO_2^-$ + $10H_2O$ | $Fe^{3+}$ | 6 | 6.3 | $1.27 \times 10^{-1}$ |
| $NH_4^+$ + 4Magnetite + $22H^+$ → $12Fe^{2+}$ +$NO_3^-$ + $13H_2O$ | $Fe^{3+}$ | 8 | 4.5 | $1.19 \times 10^{-1}$ |
| $CH_3COO^-$ + $H_2O$ → $CH_4$ + $HCO_3^-$ | C | 4 | 9.5 | $5.03 \times 10^{-2}$ |
| $NO_2^-$ + $3CHOO^-$ + $2H^+$ + $H_2O$ → $NH_4^+$ + $3HCO_3^-$ | $NO_2^-$ | 6 | 64.4 | $4.95 \times 10^{-2}$ |
| $4NO_2^-$ + $3CH_4$ + $5H^+$ + $H_2O$ → $3HCO_3^-$ + $4NH_4^+$ | $NO_2^-$ | 24 | 53.9 | $4.14 \times 10^{-2}$ |
| $CH_4$ + $2O_2$ → $HCO_3^-$ + $H^+$ + $H_2O$ | $O_2$ | 8 | 99.3 | $1.91 \times 10^{-2}$ |
| $CHOO^-$ + $6H^+$ + Magnetite → $HCO_3^-$ + $3H_2O$ + $3Fe^{2+}$ | $Fe^{3+}$ | 2 | 70.7 | $1.76 \times 10^{-2}$ |
| $CH_4$ + $1.5O_2$ → $CHOO^-$ + H2O + $H^+$ | $O_2$ | 6 | 96.1 | $1.38 \times 10^{-2}$ |
| $4NO_3^-$ + $CH_4$ → $HCO_3^-$ + $4NO_2^-$ + $H^+$ + $H_2O$ | $NO_3^-$ | 8 | 63.0 | $1.20 \times 10^{-2}$ |
| $CH_4$ + 4Magnetite + $23H^+$ → $HCO_3^-$ + $12Fe^{2+}$ + $13H_2O$ | $Fe^{3+}$ | 8 | 61.3 | $1.18 \times 10^{-2}$ |
| $NO_2^-$ + $0.5O_2$ → $NO_3^-$ | $O_2$ | 2 | 36.7 | $1.03 \times 10^{-2}$ |
| $2CH_4$ + $2O_2$ → $CH_3COO^-$ + $2H_2O$ + $H^+$ | $O_2$ | 8 | 94.5 | $9.07 \times 10^{-3}$ |
| $3Fe^{2+}$ + $0.5O_2$ + $3H_2O$ → Magnetite + $6H^+$ | $O_2$ | 2 | 70.6 | $1.54 \times 10^{-8}$ |
| $NO_2^-$ + Magnetite + $6H^+$ → $3Fe^{2+}$ + $NO_3^-$ + $3H_2O$ | $Fe^{3+}$ | 2 | -1.2 | $-3.02 \times 10^{-4}$ |
| **Scenario B. Simulated anaerobic. O2 set at 1 nM. pE = 2** | **TEA** | **# e-** | **$A_r^{e-}$ (kJ per mole e-)** | **$A_r^{kg}$ (J per Kg $H_2O$)** |
| $NO_3^-$ + $CH_3COO^-$ + $H^+$ + $H_2O$ → $NH_4^+$ + $2HCO_3^-$ | $NO_3^-$ | 8 | 61.6 | $6.55 \times 10^{-1}$ |
| $7NO_3^-$ + Pyrite + $H_2O$ → $Fe^{2+}$ + $2SO_4^{2-}$ + $7NO_2^-$ + $2H^+$ | $NO_3^-$ | 14 | 55.5 | $5.99 \times 10^{-1}$ |
| $NO_3^-$ + $4CHOO^-$ + $2H^+$ + $H_2O$ → $NH_4^+$ + $4HCO_3^-$ | $NO_3^-$ | 8 | 66.3 | $1.97 \times 10^{-1}$ |
| $CH_3COO^-$ + $H_2O$ → $CH_4$ + $HCO_3^-$ | C | 4 | 9.5 | $5.03 \times 10^{-2}$ |
| $NO_2^-$ + $3CHOO^-$ + $2H^+$ + $H_2O$ → $NH_4^+$ + $3HCO_3^-$ | $NO_2^-$ | 6 | 64.4 | $4.95 \times 10^{-2}$ |
| $4NO_2^-$ + $3CH_4$ + $5H^+$ + $H_2O$ → $3HCO_3^-$ + $4NH_4^+$ | $NO_2^-$ | 24 | 53.9 | $4.14 \times 10^{-2}$ |
| $4NO_3^-$ + $CH_4$ → $HCO_3^-$ + $4NO_2^-$ + $H^+$ + $H_2O$ | $NO_3^-$ | 8 | 62.5 | $1.20 \times 10^{-2}$ |
| $CHOO^-$ + $6H^+$ + Magnetite → $HCO_3^-$ + $3H_2O$ + $3Fe^{2+}$ | $Fe^{3+}$ | 2 | 40.1 | $9.96 \times 10^{-3}$ |
| $CH_4$ + 4Magnetite + $23H^+$ → $HCO_3^-$ + $12Fe^{2+}$ + $13H_2O$ | $Fe^{3+}$ | 8 | 30.7 | $5.90 \times 10^{-3}$ |
| Pyrite + $3.5O_2$ + $H_2O$ → $Fe^{2+}$ + $2H^+$ + $2SO_4^{2-}$ | $O_2$ | 14 | 88.2 | $1.23 \times 10^{-3}$ |
| $NH_4^+$ + 3Magnetite + $16H^+$ → $9Fe^{2+}$ + $NO_2^-$ + $10H_2O$ | $Fe^{3+}$ | 6 | 56.5 | $1.13 \times 10^{-3}$ |
| CH3COO$^-$ + $2O_2$ → $2HCO_3^-$ + $H^+$ | $O_2$ | 8 | 102.1 | $8.16 \times 10^{-4}$ |
| 2CHOOH + $O_2$ → $2HCO_3^-$ + $2H^+$ | $O_2$ | 4 | 122.4 | $2.45 \times 10^{-4}$ |
| $CH_4$ + $2O_2$ → $HCO_3^-$ + $H^+$ + $H_2O$ | $O_2$ | 8 | 92.1 | $3.68 \times 10^{-5}$ |
| $CH_4$ + $1.5O_2$ → $CHOO^-$ + $H_2O$ + $H^+$ | $O_2$ | 6 | 89.0 | $3.56 \times 10^{-5}$ |
| $2CH_4$ + $2O_2$ → $CH_3COO^-$ + $2H_2O$ + $H^+$ | $O_2$ | 8 | 87.4 | $3.49 \times 10^{-5}$ |
| $3Fe^{2+}$ + $0.5O_2$ + $3H_2O$ → Magnetite + $6H^+$ | $O_2$ | 2 | 83.9 | $3.36 \times 10^{-5}$ |
| $NO_2^-$ + $0.5O_2$ → $NO_3^-$ | $O_2$ | 2 | 29.6 | $6.36 \times 10^{-6}$ |
| $NH_4^+$ + $2O_2$ → $NO_3^-$ + $2H^+$ + $H_2O$ | $O_2$ | 8 | 35.2 | $1.41 \times 10^{-6}$ |
| $NH_4^+$ + $1.5O_2$ → $NO_2^-$ + $2H^+$ + $H_2O$ | $O_2$ | 6 | 37.1 | $6.36 \times 10^{-6}$ |
| $NH_4^+$ + 3Magnetite + $16H^+$ → $9Fe^{2+}$ + $NO_2^-$ + $10H_2O$ | $Fe^{3+}$ | 6 | -24.3 | $-4.87 \times 10^{-1}$ |
| $NH_4^+$ + 4Magnetite + 22 $H^+$ → 12 $Fe^{2+}$ +$NO_3^-$ + $13H_2O$ | $Fe^{3+}$ | 8 | -26.2 | $-6.99 \times 10^{-1}$ |
| $NO_2^-$ + Magnetite + $6H^+$ → $3Fe^{2+}$ + $NO_3^-$ + $3H_2O$ | $Fe^{3+}$ | 2 | -31.8 | $-8.14 \times 10^{-3}$ |
